# A novel nomogram individually predicting disease-specific survival after D2 gastrectomy for advanced gastric cancer

**DOI:** 10.1186/s40880-018-0293-0

**Published:** 2018-05-15

**Authors:** Wei Wang, Zhe Sun, Jing-Yu Deng, Xiao-Long Qi, Xing-Yu Feng, Cheng Fang, Xing-Hua Ma, Zhen-Ning Wang, Han Liang, Hui-Mian Xu, Zhi-Wei Zhou

**Affiliations:** 1 Department of Gastric Surgery, Sun Yat-sen University Cancer Center, State Key Laboratory of Oncology in South China, Collaborative Innovation Center for Cancer Medicine, 651 Dongfeng East Road, Guangzhou, 510060 Guangdong P. R. China; 2 Department of Surgical Oncology, The First Affiliated Hospital of China Medical University, Shenyang, 110000 Liaoning P. R. China; 3 Department of Gastric Cancer Surgery, Tianjin Medical University Cancer Hospital, Tianjin, 300000 P. R. China; 4 Department of General Surgery, Nanfang Hospital, Southern Medical University, Guangzhou, 510515 Guangdong P. R. China; 5 Department of Gastroenterology Surgery, Guangdong General Hospital, Guangzhou, 510030 Guangdong P. R. China; 6 Department of Preventive Medicine, School of Public Health, Sun Yat-sen University, Guangzhou, 510275 Guangdong P. R. China

**Keywords:** Advanced gastric cancer, Disease-specific survival, Prognostic nomogram

## Abstract

**Background:**

Few studies have shown nomograms that may predict disease-specific survival (DSS) probability after curative D2 gastrectomy for advanced gastric cancer (AGC), particularly among Chinese patients. This study sought to develop an elaborative nomogram that predicts long-term DSS for AGC in Chinese patients.

**Methods:**

A retrospective study was conducted on 6753 AGC patients undergoing D2 gastrectomy between January 1, 2000 and December 31, 2012 from three large medical hospitals in China. We assigned patients from Sun Yat-sen University Cancer Center to the training set, and patients from the First Affiliated Hospital of China Medical University and Tianjin Medical University Cancer Hospital to two separate external validation sets. A multivariate survival analysis was performed using Cox proportional hazards regression model in a training set, and a nomogram was constructed. Harrell’s C-index was used to evaluate discrimination and calibration plots were used to validate similarities between survival probabilities predicted by the nomogram model and actual survival rates in two validation sets.

**Results:**

The multivariate Cox regression model identified age, tumor size, location, Lauren classification, lymphatic/venous invasion, depth of invasion, and metastatic lymph node ratio as covariates associated with survival. In the training set, the nomogram exhibited superior discrimination power compared with the 8th American Joint Committee on Cancer TNM classification (Harrell’s C-index, 0.82 vs. 0.74; *P* < 0.001). In two validation sets, the nomogram’s discrimination power was also excellent relative to TNM classification (C-index, 0.83 vs. 0.75 and 0.81 vs. 0.74, respectively; *P* < 0.001 for both). After calibration, the nomogram produced survival predictions that corresponded closely with actual survival rate.

**Conclusions:**

The established nomogram was able to predict 3-, 5-, and 10-year DSS probabilities for AGC patients. Validation revealed that this nomogram exhibited excellent discrimination and calibration capacity, suggesting its clinical utility.

## Introduction

Despite a downward trend in incidence worldwide, gastric cancer remains both the second most prevalent cancer and the second most frequent cause of cancer-related death in China [1]. Annually, nearly one-half of the gastric cancer patients diagnosed worldwide are in China [2]. Unlike in Japan and Korea, where the majority of gastric cancers are identified at early stages [3], nearly 90% of gastric cancer patients in China are diagnosed with advanced or metastatic disease [4].

The only proven curative treatment of gastric cancer without distant metastasis is radical resection combined with regional lymphadenectomy (mostly with D2 resection in Asian countries) [5]. According to the eighth edition of the American Joint Committee on Cancer (AJCC) TNM classification, which is considered the classic cancer prediction system, the prognosis of patients with curative gastric cancer differs based on the depth of invasion and the number of metastatic lymph nodes [6].

Our previous study demonstrated that age, sex, tumor size, tumor location, and lymphatic/venous invasion (LVI) could also be considered for predicting survival probabilities [7]. Therefore, an individual predictive system beyond or combined with the TNM staging system could be useful for clinical practice and decision-making. Furthermore, accurate risk estimates may help to identify homogeneous patients at high risk to assist with clinical trial design and accrual.

Among the available decision aids, nomograms currently represent the most accurate and discriminatory tools for predicting outcomes in patients with cancer [8]. According to the strict definition, a nomogram is a graphical calculation instrument that can be based on any type of function, such as logistic regression or Cox proportional hazards regression models. The effect of the variables on the specific outcome is represented on the axes, and risk points are attributed according to the prognostic or predictive importance of the variable of interest [9].

In the past few years, nomograms have been successfully established to quantify risk by combining prognostic factors in certain malignancies [10–14]. However, few studies have used a nomogram to predict outcomes for gastric cancer patients [3, 15, 16]. The first study reporting the predictive ability of a nomogram for gastric cancer was based on a Western database [15] and was externally validated for accuracy [17]. Subsequently, studies from Korea [3] and Japan [16] were conducted to establish a nomogram based on their own databases, which mainly consisted of patients with early-stage gastric cancer and serosa-negative, locally advanced gastric cancer (AGC).

To date, no large cohort study has been conducted to establish the nomogram for the Chinese population with gastric cancer. Additionally, the demographic features, staging distributions, and treatment strategies in Chinese population differ from those in Western and Korean/Japanese populations.

In the present study, we aimed to develop and externally validate a more elaborative nomogram that predicts 3-, 5-, and 10-year disease-specific survival (DSS) probabilities in Chinese patients who have undergone curative resection for AGC. This study included a large cohort of patients from three high-capacity hospitals from southern to northern China obtained over a relatively short period of time (2000–2012). This study represented the current treatment protocol in China.

## Patients and methods

### Patient selection

Between January 1, 2000 and December 31, 2012, patients who underwent gastric cancer resection at the Department of Gastric and Pancreatic Surgery at Sun Yat-sen University Cancer Center (SYSUCC; Guangzhou, China), the Department of Surgical Oncology at the First Affiliated Hospital of China Medical University (CMU; Shenyang, China), and the Department of Gastric Cancer Surgery at Tianjin Medical University Cancer Hospital (TJMU; Tianjin, China) were included in the study. Data from patients who met the following criteria were collected: the pathologic diagnosis of primary gastric adenocarcinoma; absence of residual gastric cancer after surgery; no other synchronous malignancy; no preoperative chemotherapy; no distant metastasis; postoperative advanced disease (tumor invasion beyond the submucosa); D2 lymphadenectomy (Japanese Gastric Cancer Treatment Guideline 2010, Version 3) [18]; R0 resection (no residual macroscopic or microscopic tumor); postoperative survival of at least 3 months; and no missing data.

The DSS was defined as interval between the date of diagnosis and the date of gastric cancer-related death. Patients with no documented evidence of death were censored at the date of last follow-up. To determine prognostic values related to DSS, we collected the following variables for survival analysis and the further establishment of the nomogram: sex, age, tumor size (the longest diameter of tumor), primary site (the cardia/fundus, body, antrum, or whole stomach), Lauren classification (intestinal or diffuse), lymphatic/venous invasion (present or absent), depth of invasion (proper muscle, T2; subserosa, T3; serosa, T4a; or adjacent organ invasion, T4b), number of metastatic lymph nodes resected, and number of total lymph nodes resected. As part of our surgical procedure, accurate count of all resected lymph nodes, which were carefully retrieved by the surgeon, was recorded. The exact number of retrieved lymph nodes and detailed information about the tumors were recorded in the pathologic results.

### Follow-up

A strict disease monitoring program was conducted every 3 months for the first 2 years after surgery, every 6 months from the third year through the fifth year, and annually after 5 years. The monitoring program included abdominal ultrasonography (or computed tomography [CT] if necessary), chest radiographs, gastroduodenal endoscopy examinations, and blood examinations for carcinoembryonic antigens CA199 and CA724. Regular follow-up results were obtained from outpatient records, telephone interviews, and brief messages. This study was approved by the institutional review boards of SYSUCC, CMU, and TJMU.

The present study was approved by the ethics committees of the three hospitals and complied with the seventh version of Declaration of Helsinki in 2013.

### Construction of the nomogram

For nomogram construction and external validation, we assigned patients from SYSUCC to the training set and patients from CMU to TJMU to two separate external validation sets. A multivariate survival analysis was conducted using a Cox proportional hazards regression model without violation of the proportional hazards assumption.

The clinicopathologic characteristics of the training and validation sets were evaluated. The proportional hazards (PH) assumption and linearity assumption for continuous variables (age, tumor size, and metastatic lymph node ratio) were examined using restricted cubic splines. To maximize predictive ability, continuous variables were transformed to adequately fit the PH and linearity assumptions, if possible (based on the optimal Akaike Information Criterion value [19]). Using numerous different algorithms, the results showed that linear modeling was associated with higher predictive ability and was more appropriate for further analysis. Categorical variables were grouped based on clinical reasoning, and decisions regarding grouping were made before modeling. For the categorical variables, a log–log survival plot was used for identifying the PH assumption, and all variables were fitted to the PH assumption. Variables were selected using the forward stepwise selection method in the Cox PH regression model. Based on the predictive model with the identified prognostic factors, a nomogram was constructed for predicting 3-, 5-, and 10-year DSS probabilities.

### Validation of the nomogram

The performance of the nomogram consisted of discrimination and calibration using the external validation set. Discrimination was evaluated using a concordance index (C-index), which quantifies the probability that given two random patients, and the patient who relapses first had a higher probability of the event of interest. Harrell’s C-index, which is appropriate for censored data, was used for evaluating discrimination [20]. Calibration was performed by comparing the mean predicted survival probability with the mean actual survival rate observed by Kaplan–Meier analysis after grouping the nomogram-predicted survival probabilities by decile. Our nomogram was validated using two independent validation sets that satisfied the previously mentioned inclusion criteria. The first validation was performed using the CMU validation set, and the second validation was performed using a dataset from TJMU. All three hospitals have the same operation criteria and comprise nearly the largest gastric cancer dataset in China. Statistical significance was set as *P* < 0.05 in a two-tailed test. All statistical analyses were performed using SPSS version 20 (SPSS, Chicago, IL, USA) and R software version 2.13.2 (http://www.r-project.org) with the design and survival packages.

## Results

### Patient characteristics

**Fig. 1:**
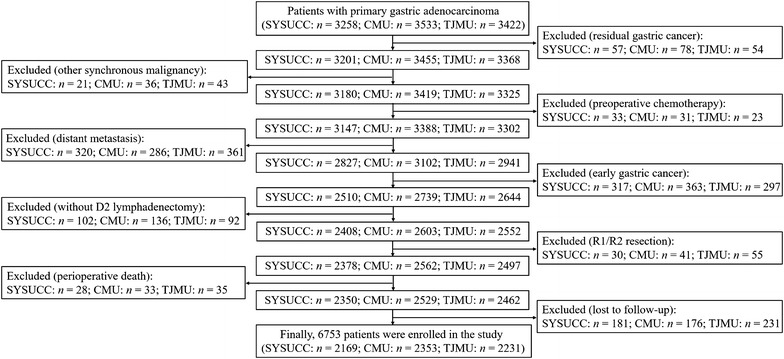
Flow chart for the study on patients with advanced gastric cancer according to inclusion and exclusion criteria in the SYSUCC, CMU, and TJMU, respectively. *SYSUCC* Sun Yat-sen University Cancer Center; *CMU* Chinese Medical University; *TJMU* Tianjin Medical University

Finally, 6753 patients were enrolled in the present study, of which 2169 were from SYSUCC, 2353 were from CMU, to 2231 were from TJMU (Fig. 1). The demographic features and clinicopathologic characteristics of the training set (SYSUCC) and validation sets (CMU and TJMU) are shown in Table 1.

**Table 1:** Demographic and clinicopathologic variables of 6753 patients with advanced gastric cancer in the training and validation sets

Variables	SYSUCC training set (*n* = 2169)	CMU validation set (*n* = 2353)	TJMU validation set (*n* = 2231)	*P* value
Age (years)^a^	59 (50–66)	60 (52–68)	59 (50–68)	< 0.001
Sex				0.049
Male	1510 (69.6)	1714 (72.8)	1576 (70.6)	
Female	659 (30.4)	639 (27.2)	655 (29.4)	
Tumor location				< 0.001
Antrum	813 (37.5)	1260 (53.5)	787 (35.3)	
Body	376 (17.3)	317 (13.5)	288 (12.9)	
Cardia/fundus	894 (41.2)	644 (27.4)	746 (33.4)	
Whole stomach	86 (4.0)	132 (5.6)	410 (18.4)	
Tumor size (cm)^a^	4.5 (3.0–6.0)	5.0 (3.5–6.5)	5.0 (3.5–7.0)	< 0.001
Lauren classification				< 0.001
Intestinal	766 (35.3)	1166 (49.6)	859 (38.5)	
Diffuse	1403 (64.7)	1187 (50.4)	1372 (61.5)	
Lymphatic/venous invasion				< 0.001
No	1917 (88.4)	1556 (66.1)	NA	
Yes	252 (11.6)	797 (33.9)	NA	
T category				< 0.001
T2	298 (13.7)	933 (39.7)	195 (8.7)	
T3	472 (21.8)	602 (25.6)	186 (8.3)	
T4a	1224 (56.4)	672 (28.6)	1673 (75.0)	
T4b	175 (8.1)	146 (6.2)	177 (7.9)	
No. of metastatic lymph nodes^a^	3 (0–8)	3 (0–8)	2 (0–7)	< 0.001
No. of lymph nodes dissected^a^	21 (13–29)	27 (18–36)	17 (11–24)	< 0.001
Metastatic lymph node ratio^a^	0.17 (0–0.43)	0.11 (0–0.33)	0.15 (0–0.46)	< 0.001

*SYSUCC* Sun Yat-sen University Cancer Center; *CMU* Chinese Medical University; *TJMU* Tianjin Medical University

^a^The terms are continues variables and their values are presented as median followed by 95% confidential interval in parentheses; other values are presented as number of patients followed by percentage in parentheses

### Establishment of nomogram

In the Cox PH regression model, age, tumor location, Lauren classification, LVI, T category, and metastatic lymph node ratio were found to be independently associated with prognosis. Table 2 shows the variables with the hazard ratios. Sex was not independently associated with prognosis. Considering the clinical utility and with the knowledge of tumour biology for prognosis, the parameter of tumor size was also included in the training and validation sets for the nomogram, even though which does not exhibit independent prognostic significance (*P* = 0.06).

**Table 2:** Selected variables according to the Cox proportional hazards regression model to construct nomogram model

Variables	Univariate analysis			Multivariate analysis		
	HR	95% CI	*P* value	HR	95% CI	*P* value
Sex	1.071	0.924–1.240	0.362			
Age (continuous)	1.018	1.011–1.024	< 0.001	1.024	1.017–1.030	< 0.001
Tumor location						
Antrum	Ref			Ref		
Body	1.494	1.214–1.837	< 0.001	1.472	1.195–1.813	< 0.001
Cardia/fundus	1.750	1.489–2.056	< 0.001	1.572	1.329–1.860	< 0.001
Whole stomach	3.597	2.674–4.839	< 0.001	1.971	1.408–2.760	< 0.001
Tumor size (continuous)	1.118	1.092–1.144	< 0.001	1.028	0.999–1.059	0.060
Lauren classification	1.350	1.164–1.565	< 0.001	1.297	1.111–1.517	0.001
Lymphatic/venous invasion	1.786	1.460–2.184	< 0.001	1.257	1.023–1.544	0.030
T category						
T2	Ref			Ref		
T3	2.223	1.597–3.096	< 0.001	1.581	1.132–2.209	0.007
T4a	3.419	2.545–4.594	< 0.001	2.204	1.631–2.979	< 0.001
T4b	5.432	3.853–7.660	< 0.001	2.929	2.051–4.183	< 0.001
Metastatic lymph node ratio (continuous)	8.423	6.798–10.436	< 0.001	6.815	5.411–8.582	< 0.001

*HR* hazard ratio; *CI* confidential interval

**Fig. 2:**
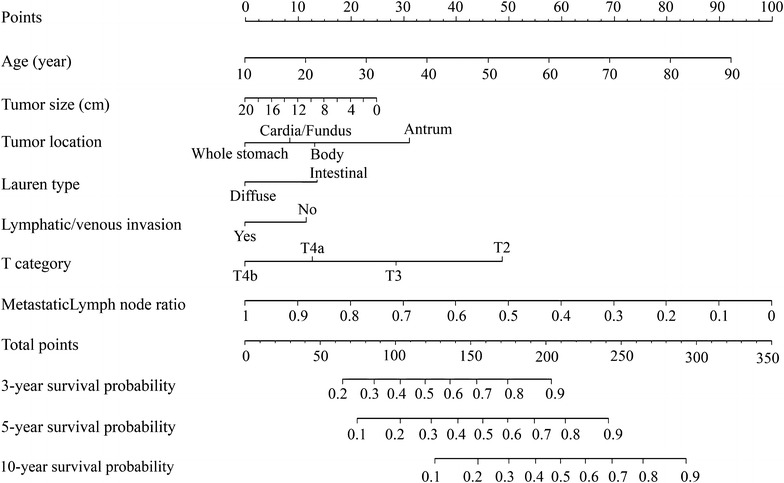
A nomogram predicting 3-, 5-, and 10-year disease-specific survival probabilities of patients after D2 gastrectomy for advanced gastric cancer in the Sun Yat-sen University Cancer Center training set. The nomogram is used by summing the points identified on the point scale for all variables. The total points projected on the bottom scales indicate the probabilities of 3-, 5-, and 10-year survival

Figure 2 illustrates the predictive nomogram model established for 3-, 5-, and 10-year DSS based on the selected variables in the training sets. A patient’s individual survival probability was easily calculated by summing the points for each selected variable. In our nomogram model, we attempted to utilize both continuous and categorical variables to fit the model based on the Akaike Information Criterion value. In the best predictive model, we found that using continuous variables was more suitable than converting them into categorical variables, based on the highest C-index value. We compared the discrimination of the nomogram with that of the eighth AJCC TNM classification in the SYSUCC training set. Nomogram discrimination was 0.82 (95% confidential interval [CI], 0.79–0.85), which was superior to that of the eighth AJCC TNM classification (C-index, 0.74; 95% CI, 0.72–0.77; *P* < 0.001).

### Validation and performance of the nomogram

In the CMU and TJMU validation sets, discrimination was also better for the nomogram than for AJCC TNM staging (C-index, 0.83 [95% CI, 0.80–0.86] vs. 0.75 [95% CI, 0.72–0.77] and 0.81 [95% CI, 0.78–0.83] vs. 0.74 [95% CI, 0.71–0.76], respectively; *P* < 0.001 for both comparisons).

**Fig. 3:**
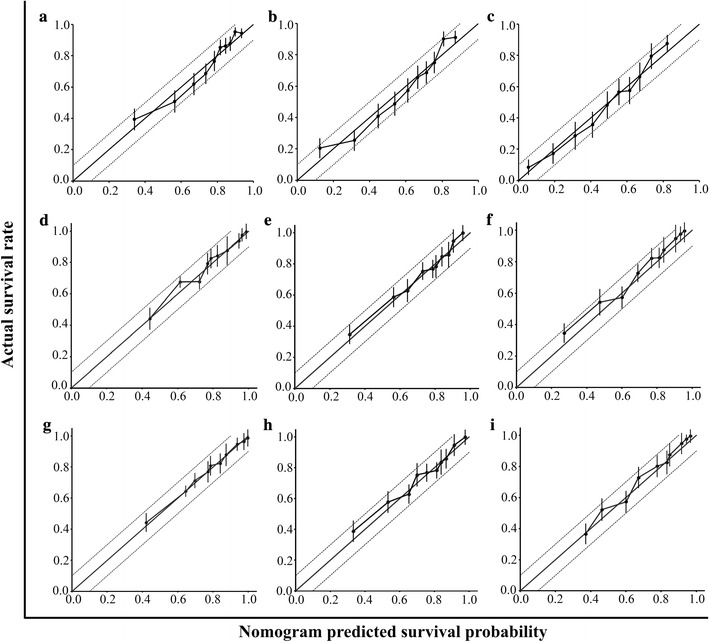
Calibration of the nomogram in the CMU and TJMU validation sets. The x-axis represents nomogram-predicted survival probabilities, and the y-axis represents actual survival rates, with 95% confidential intervals measured by Kaplan–Meier analysis. All predictions lie within a 10% margin of error (within the dashed lines). **a**–**c** represents the 3-, 5-, and 10-year survival of the SYSUCC training set; **d**–**f** represents the 3-, 5-, and 10-year survival of the CMU validation set; and **g**–**i** represents the 3-, 5-, and 10-year survival of the TJMU validation set. *SYSUCC* Sun Yat-sen University Cancer Center; *CMU* Chinese Medical University; *TJMU* Tianjin Medical University

**Fig. 4:**
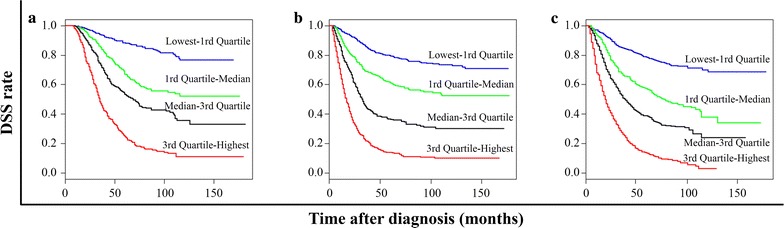
Kaplan-Meier curves according to quartiles of nomogram point. **a** SYSUCC; **b** CMU; **c** TJMU. *SYSUCC* Sun Yat-sen University Cancer Center; *CMU* Chinese Medical University; *TJMU* Tianjin Medical University

To validate similarities between survival probabilities predicted by the nomogram model and actual survival rates, a calibration plot was generated (Fig. 3). The results demonstrate that the actual 3-, 5-, and 10-year survival rate corresponded closely to the predicted survival probabilities, within a 10% margin of error represented by dotted lines, in both the training set and the validation sets. Furthermore, the Kaplan–Meier curves according to quartiles of nomogram point illustrated excellent discriminatory ability between each quartile in the SYSUCC training set and those in the CMU and TJMU validation sets (Fig. 4).

## Discussion

In the present study, we established and externally validated a nomogram model for predicting DSS after D2 gastrectomy for AGC based on a large Chinese cohort. This nomogram was more significantly predictive than the eighth edition AJCC staging, with a C-index of 0.82 and good calibration. Compared with previous studies [3, 15, 16], this study included a larger number of patients with AGC (*n* = 6753), and the nomogram had a higher precision. Furthermore, our nomogram considered the unique characteristics of the patient population, thus addressing discrepancies in previous models regarding demographic features, stage distribution, and treatment strategies of US, Korean, or Japanese gastric cancer patients.

In contrast to the previous nomogram based on US [15], Korean [3], or Japanese [16] studies, sex was excluded from the nomogram in our present study. This is interesting because female gastric cancer patients had better prognoses in both the univariate and multivariate analyses in all of the previously mentioned databases. However, in our study cohort, the superior survival rate of female patients was insignificant (*P* = 0.360). We believe that this finding represents one of the most important differences in demographic features and that it may be due, in part, to disadvantages in terms of socioeconomic status of the female population in China.

In our nomogram, Lauren classification was included because of its independent prognostic effect, which corresponds with the US [15] database but not the Korean [3] and Japanese [16] databases. This variable was excluded from the Korean database due to a large percentage of missing data and was replaced with macroscopic and histologic types in the model from the Japanese database. Previous research demonstrated a relationship between Lauren classification and the effect of trastuzumab in gastric cancer patients [21]. Therefore, because one purpose of the nomogram was to guide clinical practice, we chose to include the Lauren classification as an important predictive variable in our nomogram.

One important improvement of our nomogram is that we chose to include metastatic lymph node ratio, which refers to the number of metastatic lymph nodes resected divided by the number of total lymph nodes resected. In our previous studies, we successfully verified that the metastatic lymph node ratio had theoretical and practical advantages for decreasing the staging migration phenomenon and improving prognostic prediction [22–24]. However, based on the independent datasets from the three hospitals included in the present study, the optimal cutoff values of metastatic lymph node ratio for prediction were different. In contrast to models based on the TNM staging system, the predictive model based on the nomogram can directly use a continuous variable without requiring this variable to be converted into a categorical variable. We believe that this model may be considered a general predictive tool for future clinical use. Another important improvement of our nomogram is the use of continuous variables for the establishment of the model. In the present study, we tested various methods of treating or converting continuous variables such as age, tumor size, and metastatic lymph node ratio. We determined that the use of continuous variables resulted in the most precise predictions.

The accuracy of a predictive model refers to the ability of the model to discriminate between patients with or without the outcome of interest, which is demonstrated by the C-index for censored data. In our nomogram model, the C-index values were high: 0.82 in the training set and 0.83 and 0.81 in validation sets. Furthermore, a model’s accuracy indicates not only the overall ability to predict the outcome of interest but also the ability to predict the outcome of interest in specific patient groups or according to risk level. For this purpose, calibration plots should be obtained for both internal and external data. Unlike previous studies, our study investigated all three independent datasets.

We hypothesize that the inclusion of complete and precise data (including all follow-up data), the conversion of continuous data, and the stage distribution contributed to that our study has a higher C-index value compared with previous studies [3, 15, 16]. In our research dataset, we excluded all cases with missing data to improve the precision of the predictive model. For the same reason, we tried various methods to convert the continuous variables (including age, tumor size, and metastatic lymph node ratio) to maximize the predictive ability of the model. The predictive ability increased gradually from 0.76 to 0.82. Furthermore, the stage distribution was distinct from that observed in previous studies [3, 15, 16], for example, in studies using a dataset including early-stage gastric cancers (the US and Korean datasets) or excluding locally AGC (the Japanese dataset). On the other hand, with advanced disease, the demand for precise prediction of survival probability becomes increasingly important for clinical decisions regarding the use of adjuvant chemotherapy or chemoradiotherapy. For this reason, we think that precise prediction tools are more important in AGC patients than in patients with relatively early-stage diseases.

This study has some limitations. The main limitation of the present study was that adjuvant therapy, such as chemotherapy or chemoradiotherapy, was not included as a variable in the predictive model. In the multivariate analysis using the Cox regression model, adjuvant therapy did not appear to be important for prognosis. In clinical practice in China, patients with stage II or III disease usually receive adjuvant chemotherapy with a relatively uniform protocol including 5-fluorouracil- and/or platinum-based therapeutics. In some patients with high-risk recurrent diseases, adjuvant chemoradiotherapy is also recommended. Therefore, adjuvant therapy was not included as a variable in the present study. Another limitation was that our external validation set was based on the Chinese population. This is a large and multicenter database on Chinese patients has been used to establish such a model. In addition, the baseline parameters at the three hospitals were different because the patients from each hospital come from specific regions of China and thus have comparatively different demographics. However, despite these differences, the nomogram demonstrated great accuracy as shown by the C-indexes (SYSUCC: 0.82; CMU: 0.83; and TJMU: 0.81). Whether this predictive model is suitable for Western, US, or other Asian countries is unknown. Therefore, in future studies, it will be necessary to validate this model using data from other countries.

In conclusion, in the present study, we established a nomogram for precisely and individually predicting DSS for Chinese patients with AGC who underwent radical resection with D2 lymphadenectomy based on databases from multiple centers. Its superiority to the traditional TNM staging system may enhance its clinical use in the near future.
